# Acupuncture on GB34 activates the precentral gyrus and prefrontal cortex in Parkinson’s disease

**DOI:** 10.1186/1472-6882-14-336

**Published:** 2014-09-15

**Authors:** Sujung Yeo, Il-Hwan Choe, Maurits van den Noort, Peggy Bosch, Geon-Ho Jahng, Bruce Rosen, Sung-Hoon Kim, Sabina Lim

**Affiliations:** Research Group of Pain and Neuroscience, WHO Collaborating Centre, East–west Medical Research Institute, Kyung Hee University, Seoul, Republic of Korea; College of Oriental Medicine, Kyung Hee University, Seoul, Republic of Korea; Free University of Brussels, Brussels, Belgium; Donders Institute for Brain, Cognition and Behaviour, Radboud University Nijmegen, Nijmegen, The Netherlands; College of Medicine, Kyung Hee University, Seoul, Republic of Korea; Athinoula A. Martinos Center for Biomedical Imaging, Department of Radiology, Massachusetts General Hospital, Harvard Medical School, Boston, USA

**Keywords:** Functional magnetic resonance imaging, Parkinson’s, Healthy, Patient

## Abstract

**Background:**

Acupuncture is increasingly used as an additional treatment for patients with Parkinson’s disease (PD).

**Methods:**

In this functional magnetic resonance imaging study, brain activation in response to acupuncture in a group of 12 patients with PD was compared with a group of 12 healthy participants. Acupuncture was conducted on a specific acupoint, the right GB 34 (Yanglingquan), which is a frequently used acupoint for motor function treatment in the oriental medical field.

**Results:**

Acupuncture stimulation on this acupoint activates the prefrontal cortex, precentral gyrus, and putamen in patients with PD; areas that are known to be impaired in patients with PD. Compared with healthy participants, patients with PD showed significantly higher brain activity in the prefrontal cortex and precentral gyrus, especially visible in the left hemisphere.

**Conclusions:**

The neuroimaging results of our study suggest that in future acupuncture research; the prefrontal cortex as well as the precentral gyrus should be treated for symptoms of Parkinson’s disease and that GB 34 seems to be a suitable acupoint. Moreover, acupuncture evoked different brain activations in patients with Parkinson’s disease than in healthy participants in our study, stressing the importance of conducting acupuncture studies on both healthy participants as well as patients within the same study, in order to detect acupuncture efficacy.

**Trial registration:**

KCT0001122 at cris.nih.go.kr (registration date: 20140530)

**Electronic supplementary material:**

The online version of this article (doi:10.1186/1472-6882-14-336) contains supplementary material, which is available to authorized users.

## Background

Acupuncture is an important modality of complementary medicine [1, 2] and numerous researchers have studied its effects [3–6]. In acupuncture studies, functional magnetic resonance imaging (fMRI) is the most commonly applied method of functional neuroimaging, because it can indirectly measure brain activity and functional changes of the brain without harmful radiation and invasive procedures [5, 7–11].

To measure the effects of acupuncture with fMRI, studies have been conducted on healthy participants [12–15], indicating that each acupoint has its own specific effect, and on patients with diseases, showing that acupuncture is able to improve the symptoms of patients [16–18]. However, to our knowledge, there has so far been no fMRI study that directly measures and compares brain activity in response to acupuncture stimulating manipulation in healthy participants to those of patients with Parkinson’s disease (PD). Because acupuncture is being used to treat patients with PD [19], it is important to conduct an fMRI study on both patients with PD and healthy participants, to better understand the effects of acupuncture on patients with PD, and possibly look for evidence of its functional mechanism. Moreover, compared to healthy participants, abnormal brain activation was reported in the patients with PD [20], therefore it is important to investigate whether acupuncture stimulation on GB 34 has an effect on brain areas that are known to show dysfunction.

PD is one of the most common neurodegenerative disorders. Its characteristic features include rest tremor, rigidity, and bradykinesia [21–23]. Complementary and alternative therapy is being used more often as a complementary treatment to Western medicine for patients with PD [24–27]. As many as 40% of patients with PD use some form of complementary medicine during the course of their illness [28], and among these, acupuncture is popular [1]. Acupuncture has been reported to have a neuroprotective effect in PD animal models [4, 29–32]. In particular, the acupoint GB 34 was reported to lead to significant improvements in patients with PD [1, 33, 34] and significant neuroprotective effects in PD animal models [6, 35, 36].

Based on the literature, we hypothesize that acupuncture stimulation on GB 34 has an effect on brain areas that are known to show dysfunction due to nigral dopamine depletion: the prefrontal cortex [37], precentral gyrus [38], thalamus [39], globus pallidus [40], caudate [41], and putamen [38]. Moreover, our second hypothesis is that a significant difference in brain activity in patients with PD compared with healthy participants will be found.

## Methods

### Ethics statement

This study has been approved by the Clinical Research Information Service (CRiS). The approved number is “KCT0001122” and the topic is titled “*Development of Acupuncture Treatment for patient with Parkinson’s disease*”. Participants were given written information and verbal explanation concerning the study.

### Participants

Twenty-four volunteers participated following written informed consent according to the institutional guidelines of IRB and in accordance with the Declaration of Helsinki. 12 participants were idiopathic patients with PD (mean age = 53.5 years (range: 38–72), 6 males), whereas the other 12 volunteers were healthy participants, matched for age (mean age = 55.9, (range: 35–71)) and gender (6 males). Patients with atypical parkinsonian disorder, other neurological or major medical conditions (e.g. head injury, stroke) or current psychiatric problems were excluded from the study. All healthy volunteers and patients with PD were familiar with acupuncture stimulation to control for the acupuncture experience in the study. PD participants were diagnosed with clinically definite idiopathic PD by a neurologist from the Kyung Hee Medical Hospital. All were studied in the “off” condition; 12 hours after all anti-parkinsonian drugs had been withheld. Four patients with PD showed right-onset, 5 patients showed left-onset and 3 patients showed bilateral-onset. All had Hoehn and Yahr stage [42] 1, 2, or 2.5. The mean Unified Parkinson’s Disease Rating Scale (UPDRS) [43] motor score was 7.8 (*SD* = 3.9). All patients with PD were right-handed as verified by the Edinburg Handedness Inventory [44]; their mean score was 99.6% (*SD* = 1.4). The average duration of disease was 2.7 years. Healthy participants were without any neurological or psychiatric history and were all right-handed as verified by the Edinburgh Handedness Inventory [44]; their mean score was 100% (*SD* = 0, Additional file 1: Table S1).

### Acupuncture

An eastern medical doctor with more than 5 years of clinical experience conducted acupuncture on the right GB 34 (Yanglingquan) (Additional file 2: Figure S1), according to the WHO Standard Acupuncture Point Locations and according to the STRICTA norms [45]. This acupoint, which is on the fibular aspect of the leg, in the depression anterior and distal to the head of the fibula, was chosen because it is reported that it leads to significant neuroprotective effects in PD animal models [6, 46] and significant improvements in patients with PD [34, 47]. For verum acupuncture stimulation (ACUP), the needle (0.25 × 40 mm, Dong Bang Acupuncture Inc. Seoul, Korea) was manually inserted into the right GB 34 to a depth of approximately 1.0 cm. During ACUP, there were blocks in which the needle was “stimulated”, for which the needle was not only inserted into the skin, but also rotated bidirectionally at 1 Hz and there were blocks in which the needle was “not stimulated”, for which the needle was only inserted into the skin and then left in place. For sham acupuncture stimulation (SHAM) [48], a blunt type needle was used [47]. In contrast to ACUP, the blunt type needle was not inserted into the skin. With the exception of this, the same paradigm as for ACUP was used.

### Experimental design and procedure

The participants received general instructions of the experiment, followed by more detailed instructions for the specific task. First, participants signed the informed consent form, according to institutional guidelines of the Human Research Committee of Kyung Hee Medical Hospital. Then, all participants completed the Edinburgh Handedness Inventory [44], Hoen and Yahr stage [42], and were reminded of the specific task instructions of the fMRI experiment. They were instructed not to move their bodies and heads [49]. A block design was used. The scanning experiment started with the SHAM condition with 5 minutes durations. Then, anatomical reference images were obtained for more than 15 minutes, followed by the ACUP condition with 5 minutes durations (Additional file 3: Figure S2). Following the ACUP and SHAM scannings, participants rated the intensity of the sensation they felt [48]. Finally, after the fMRI experiment, participants conducted the UPDRS [43], the Korean Mini-Mental State Examination [50] and the Beck Depression Inventory [51].

### MRI data acquisition

A Philips 3.0 T MRI system (Philips, Netherlands) equipped for echo planer imaging (EPI) was used for data acquisition. For each participant, 270 contiguous EPI functional volumes (time repetition [TR] = 2000 ms, time echo [TE] = 35 ms, flip angle = 90°, slice thickness = 4.5, matrix = 96 × 128, field of view [FOV] = 230 × 182 × 135 mm, acquisition voxel size = 2.4 × 2.4 × 4.5 mm) were collected. During scanning, participants remained in the supine position with their heads immobilized by cushioned supports. They wore ear plugs throughout the experiment to attenuate MRI gradient noise. Moreover, they were instructed to rest with their eyes closed and not to move. For spatial normalization and localization, a high resolution T1-weighted anatomical image was then acquired using a magnetization prepared gradient echo sequence (time repetition [TR] = 9.9 ms, time echo [TE] = 4.6 ms, flip angle = 90°, slice thickness = 1 mm, matrix = 236 × 240, field of view [FOV] = 235 × 235 × 196 mm, acquisition voxel size = 1 × 1 × 1 mm).

### MRI data analysis

fMRI data were analyzed using SPM5. The first five volumes of each participant’s dataset were discarded to allow for T1 equilibration. The functional EPI-blood oxygenation level dependant (BOLD) images were realigned, and the subject-mean functional MR images were co-registered with the corresponding structure MR images. These images were spatially normalized and transformed into a common space, as defined by the SPM Montreal Neurological Institute (MNI) T1 template. The functional EPI-BOLD images were spatially smoothed with a Gaussian kernel of 8 mm FWHM (full-width half-maximum).

The fMRI data were then statistically analyzed using the general linear model and statistical parametric mapping of SPM5. At the first-level, single-subject fixed effect analyses were conducted. A model with the experiment conditions was tested in each participant’s data separately. For second-level analysis, the generated contrast images for the main effects were assessed by conducting a one-sample *t*-test. The comparison between healthy participants and patients with PD was assessed by conducting a two-sample *t*-test. Significant differences were accepted at a threshold of corrected cluster level *P* < 0.05 [52].

All local maxima are reported as Talairach coordinates. In addition, the cluster size and the peak *t* value of areas of significant increase are given. Relevant anatomical landmarks and Brodmann areas were identified using GingerALE (http://www.brainmap.org) and analyzed step by step using Talairach Client (http://www.talairach.org). The regions of interest (ROI) were fixed using Automated Anatomical Labeling. The activated volume of ROI of each region was measured using MRIcroN. The volume comparison between ACUP and SHAM in each group was assessed by conducting a paired-*t* test (SPSS Inc., Chicago, IL, USA).

## Results

### Psychophysical responses

The intensity of sensations measured by an average score was reported on a scale from 0 (denoting no sensation) to 10 (denoting an unbearable sensation between patients with PD and healthy participants during ACUP and SHAM). The average stimulus intensities (mean ± *SD*) were approximately similar during ACUP of patients with PD (2 ± 2.4), SHAM of patients with PD (1.3 ± 2.0), ACUP of healthy participants (1.4 ± 2.1), and SHAM of healthy participants (0.9 ± 1.9); no significant statistical differences were found.

### PD related brain activation induced by ACUP and SHAM in healthy participants and patients with PD

In healthy participants, the right putamen, left and right thalamus, right caudate body, right lateral globus pallidus, exhibited significantly higher brain activation than other brain areas during ACUP. During SHAM, healthy participants showed significant brain activation in the right putamen, right precentral gyrus, right inferior, frontal gyrus, right putamen and right thalamus.

**Figure 1:**
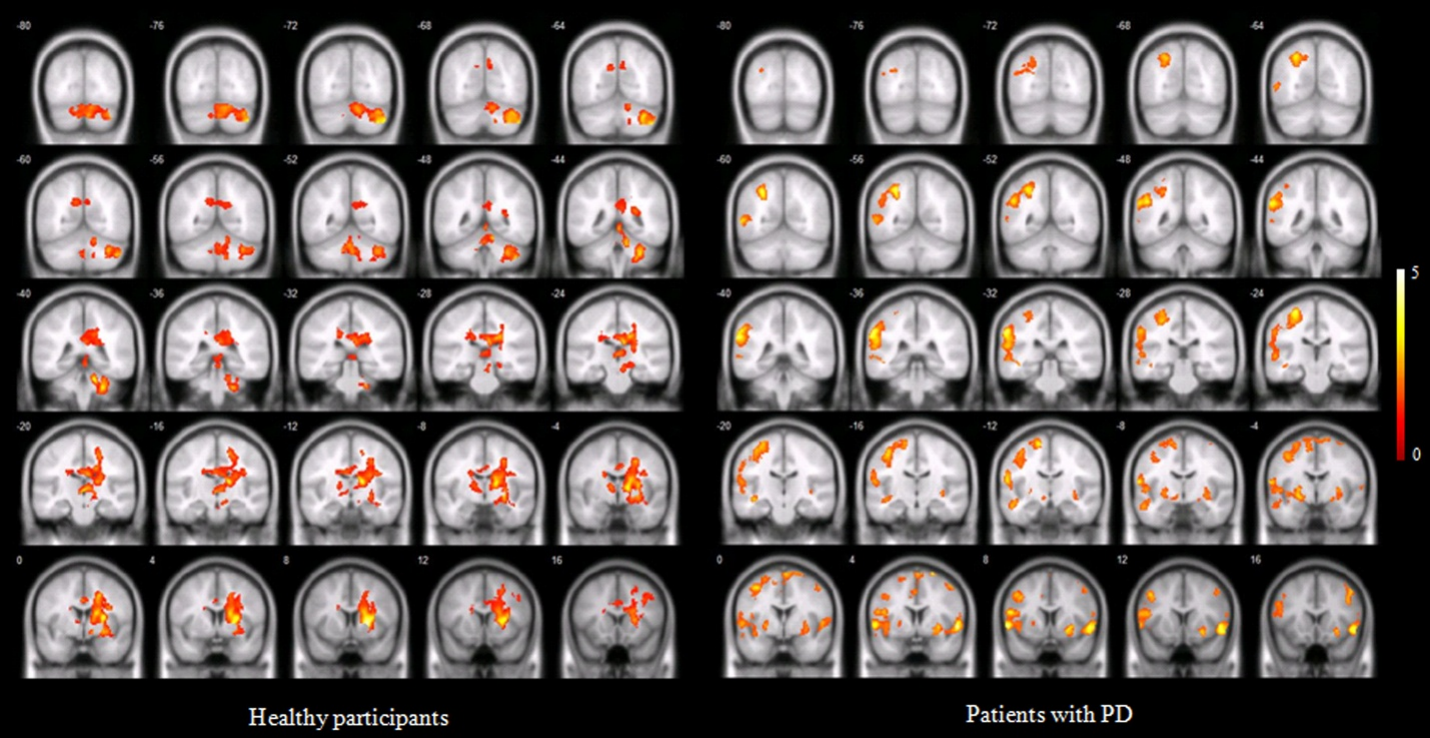
**Brain activation among healthy participants and patients with PD during acupuncture stimulation and sham stimulation (one-sample** ***t*** **-test; with corrected cluster level** ***P*** **< 0.05).** The bar is the *t* value.

In patients with PD, the right superior frontal gyrus, left middle frontal gyrus, left and right inferior frontal gyrus, left and right precentral gyrus and left and right putamen exhibited significantly higher brain activation than other brain areas during ACUP. During SHAM, the left superior frontal gyrus, left middle frontal gyrus, left and right inferior frontal gyrus, left and right precentral gyrus and right putamen exhibited significantly higher brain activation than other brain areas in patients with PD (Figure 1, Table 1 and Additional file 4: Table S2).

**Table 1:** **PD related brain activation induced by ACUP and SHAM in healthy participants and patients with PD**

	SHAM Healthy		ACUP Healthy		SHAM PD		ACUP PD	
	L	R	L	R	L	R	L	R
Prefrontal cortex		o			o	o	o	o
Precentral gyrus		o			o	o	o	o
Thalamus		o	o	o				
Globus pallidus				o				
Caudate				o				
Putamen		o		o		o	o	o

### Comparison of brain activation between healthy participants and patients with PD during ACUP

Healthy participants showed significantly higher brain activation in the right middle frontal gyrus, right hippocampus, left and right caudate body, left and right caudate tail, left thalamus, right putamen, right insula and right cingulate gyrus than patients with PD.

Moreover, patients with PD showed significantly higher brain activation in the left inferior frontal gyrus, left precentral gyrus, left precuneus, left postcentral gyrus, left superior temporal gyrus, left middle temporal gyrus, left inferior temporal gyrus, left insula, left claustrum and left fusiform gyrus than healthy participants (Additional file 5: Figure S3 and Table 2).

**Table 2:** **Comparisons of neural responses between patients with PD and healthy participants during acupuncture stimulation**

			Healthy > Patients with PD				Healthy < Patients with PD			
Cerebral area			Coordinates anatomical location			Statistical values	Coordinates anatomical location			Statistical values
Brain region	L/R	Brodmann area	***x***	***y***	***z***	***t***value	***x***	***y***	***z***	***t***value
*Frontal lobe*										
Middle frontal gyrus	Right	6	21	5	48	3.19	-	-	-	-
Inferior frontal gyrus	Left	45/47	-	-	-	-	-32	29	-1	4.47
Precentral gyrus	Left	4/6	-	-	-	-	-28	-29	57	5.36
*Parietal lobe*										
Precuneus	Left	7	-	-	-	-	-26	-59	36	5.37
Postcentral gyrus	Left	2/3/40	-	-	-	-	-46	-19	43	5.36
*Temporal lobe*										
Hippocampus	Right		32	-35	0	4.63	-	-	-	-
Superior temporal gyrus	Left	22/38/41	-	-	-	-	-51	-22	3	4.69
Middle temporal gyrus	Left	37	-	-	-	-	-40	-60	5	4.39
Inferior temporal gyrus	Left	19	-	-	-	-	-44	-52	2	3.99
*Sub-lobar*										
Caudate body	Right		15	-29	27	5.04	-	-	-	-
Caudate tail	Right		25	-35	21	3.96	-	-	-	-
Putamen	Right		25	3	16	3.98	-	-	-	-
Insula	Right	13	30	-15	27	3.41	-	-	-	-
Caudate body	Left		-16	-23	27	4.11	-	-	-	-
Caudate tail	Left		-18	-30	26	3.92	-	-	-	-
Thalamus	Left		1	-19	4	3.88	-	-	-	-
Insula	Left	13	-	-	-	-	-45	-5	5	4.81
Claustrum	Left		-	-	-	-	-31	19	0	4.17
*Limbic lobe*										
Cingulate gyrus	Right	23/24/31/32	23	-13	39	4.33	-	-	-	-
*Occipital lobe*										
Precuneus	Left	31	-	-	-	-	-27	-71	21	4.34
Fusiform gyrus	Left	19	-	-	-	-	-21	-66	-8	3.99
Cluster size			9920				18776			

The table describes the location of the peak voxel and the corresponding brain regions and Brodmann areas comprised by the cluster. Results are reported if cluster level corrected *P* < 0.05. The voxel size is 2.4 × 2.4 × 4.5 mm. “Healthy participants > Patients with PD” indicates more activated neural responses of healthy participants compared to patients with PD. “Healthy participants < Patients with PD” indicates more activated neural responses of patients with PD compared to healthy participants (two-sample *t* test; with corrected cluster level *P* < 0.05).

### Volume of the prefrontal cortex and precentral gyrus activated by ACUP and SHAM

**Figure 2:**
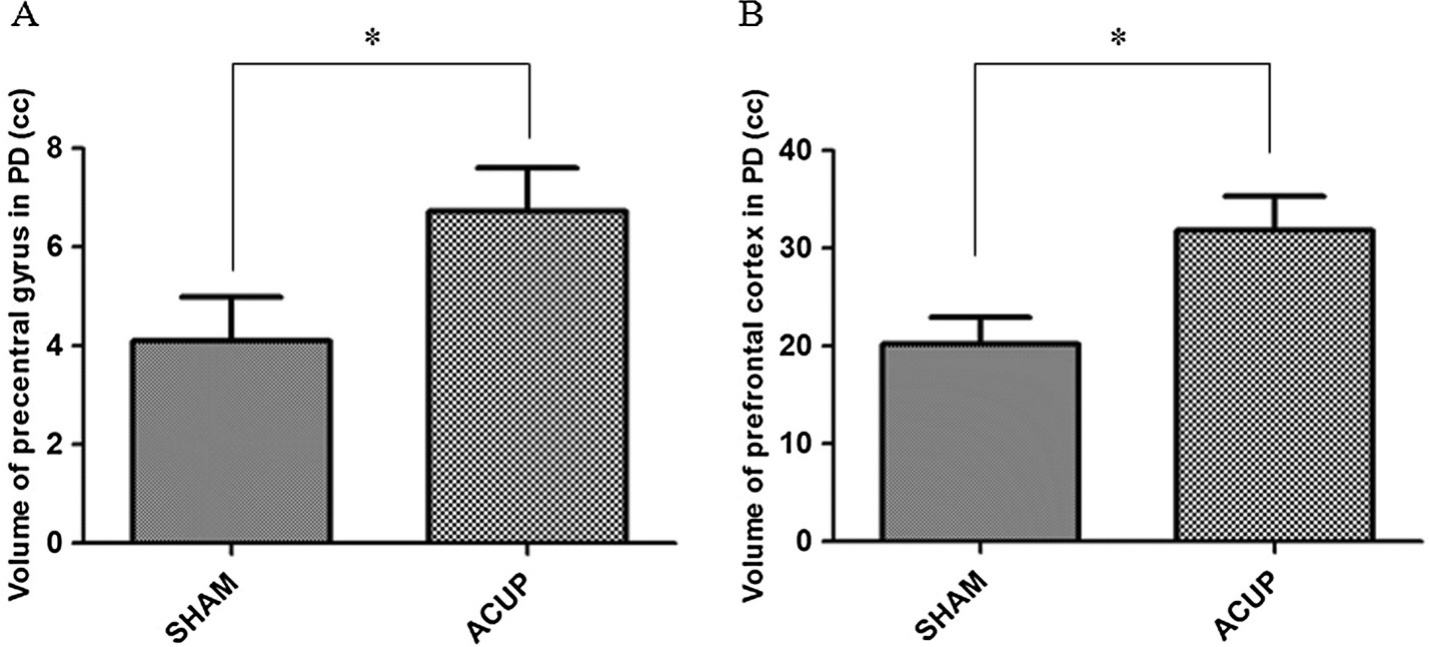
**Volume of areas activated by ACUP and SHAM.** ‘**A**’ is for the precentral gyrus and ‘**B**’ is for the prefrontal cortex (paired-*t* test; *P < 0.05).

Volume of the prefrontal cortex and precentral gyrus induced by ACUP were significantly bigger than that by SHAM (Figure 2).

### Common areas activated on both patients with PD and healthy participants during ACUP and SHAM

**Figure 3:**
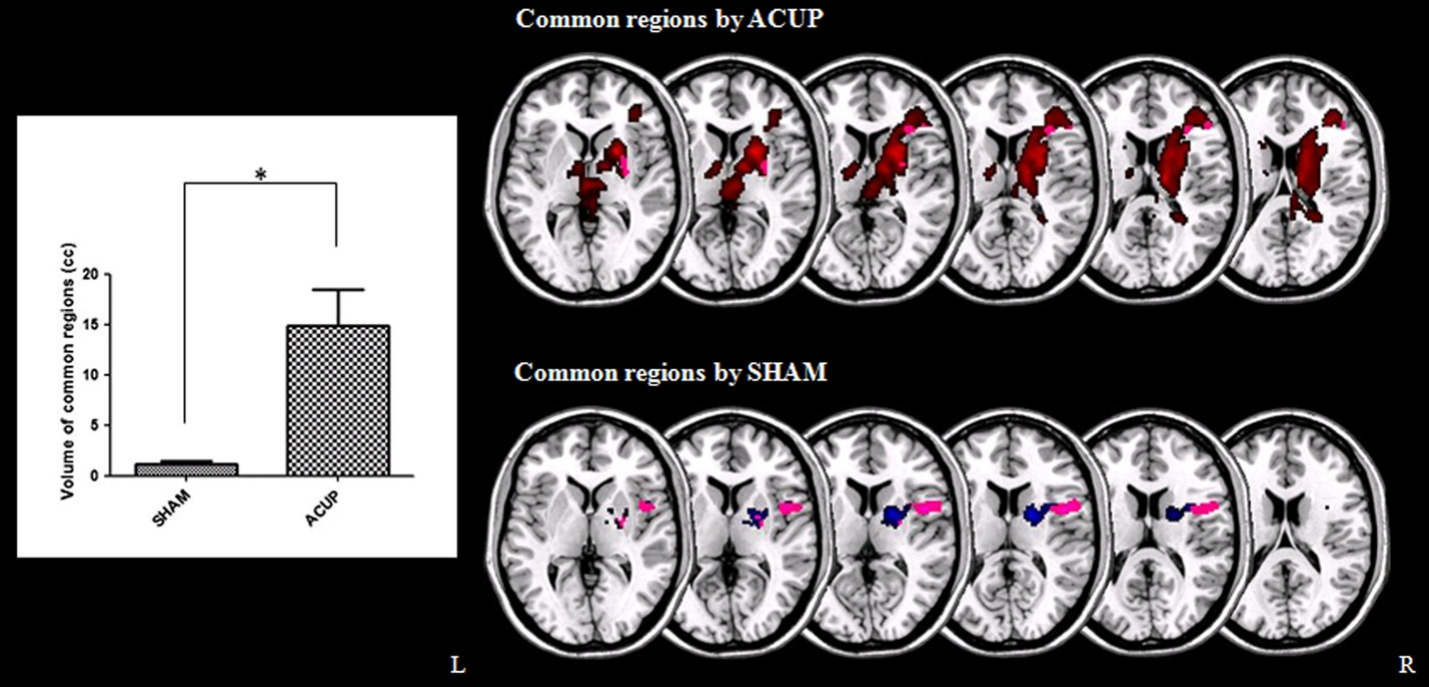
**Common areas activated on both patients with PD and healthy participants during ACUP and SHAM.** Left graph shows the significant difference in common region volume activated by ACUP and SHAM. *P < 0.005.

The volume of common areas which were activated by ACUP was significantly larger than that by SHAM (Figure 3).

## Discussion

In this fMRI study, brain activation due to acupuncture in a group of healthy participants and in a group of patients with PD was investigated. We hypothesized that ACUP on GB 34 has an effect on brain areas that are known to show dysfunction due to nigral dopamine depletion. Moreover, our second hypothesis was that a difference in brain activity would be identified in patients with PD when compared with healthy participants.

In line with our first hypothesis, our neuroimaging results show that ACUP on GB 34, among other areas, indeed activated the prefrontal cortex and the precentral gyrus in patients with PD, but not in healthy participants (Figure 1 and Table 1). Moreover, the putamen was activated in both groups. The prefrontal cortex [37], precentral gyrus [38], and putamen [38] are all known to be affected by PD. In a simple finger-tapping task, acupuncture at GB34 showed a significant improvement of motor function on the affected hand before and after ACUP on GB34 (13.6%; *P* < 0.05) [47], and led to increased activation of the putamen and the primary motor cortex in patients with PD [47]. The precentral gyrus is a part of the primary motor cortex that contains large neurons, which connect to the muscle [53]. The putamen is interconnected with so many other structures, it works in conjunction with them to control many types of motor skills such as motor learning, performance [54], preparation [55] and movement sequences [56]. Local impairments in the putamen may affect the whole corticostriatal network [57]. In particular, brain activation of the left putamen was only shown following ACUP in patients with PD. It seems that acupuncture, which is known to have an effect on the dopamine system [58], affects these dysfunctional corticostriatal networks.

Our results following ACUP also exhibited significant brain activation in the right superior and inferior frontal gyrus and left middle and inferior frontal gyrus among the prefrontal cortex (Additional file 5: Table S2). Consistent with previous studies, our results also confirmed that ACUP activated areas related to corticostriatal networks, which are impaired in patients with PD.

In addition, the thalamus [39] and the globus pallidus [40] were, according to our first hypothesis, also activated after ACUP on GB 34; however, this was only the case in healthy participants. More specifically, in healthy participants, it was found that almost all brain activation occurred in the basal ganglia. The right putamen, left and right thalamus, right caudate, right insula, and right lateral globus pallidus exhibited significantly higher brain activation than other brain areas during ACUP (Table 1).

Consistent with previous studies, differences of brain activity between ACUP and SHAM in healthy participants were observed. ACUP on left GB34 in healthy participants activated the right putamen, caudate body, claustrum, thalamus, cerebellum, as well as the left caudate body, thalamus, and cerebellum, all which are related to motor function [59], whereas very few areas were activated when SHAM was given [60]. Through these results, we confirmed that our experimental methods were clear and resulted in our acceptance of the results. Based on the results of healthy participants, we could find abnormal responses to stimulations in patients with PD.

As can be seen in Figure 1, in patients with PD, regions of the brain activated after ACUP were different with those after ACUP in healthy participants. Patients with PD have dysfunction due to nigral dopamine depletion, which may cause abnormal activation of the basal ganglia compared with healthy participants [61–63]. Previous studies have shown that compared with healthy participants, increased fMRI signals were found in patients with PD during motor tasks [63], ankle movement [64] and motor responses [61], because they are likely to participate in the same putative attempt by the dopamine-denervated brain to recruit parallel motor circuits to overcome the functional deficit of the striatocortical motor loops. In our results, areas activated in SHAM, including the paracentral lobule, superior frontal gyrus, inferior parietal lobule, superior parietal lobule, precuneus, middle temporal gyrus, inferior temporal gyrus, claustrum, insula and middle occipital gyrus, all disappeared following ACUP. Instead of that, the left putamen was activated following acupuncture. It is possible that these areas became activated to compensate for nigral dopamine depletion in patients with PD and that ACUP may calm these areas down to normal activity levels, as observed in healthy participants.

With respect to our second hypothesis, a comparison of brain activation results (Table 2) showed that ACUP indeed evoked different brain activation in patients with PD than in healthy participants. Interestingly, patients with PD showed significantly higher brain activation in the prefrontal cortex and precentral gyrus, especially in the left hemisphere. Because acupuncture in our study was conducted on the right leg, brain activation can be expected to be greater in opposite brain areas. Moreover, note that the prefrontal cortex and precentral gyrus are known to be affected by PD [37, 38]. Our neuroimaging results seem to indicate that the effects of acupuncture treatment are visible in these areas in patients with PD, but not in healthy participants.

However, it remains unclear why the effect of acupuncture on GB 34 is visible in the prefrontal cortex and precentral gyrus and not in the thalamus, globus pallidus, and caudate, which are areas that are also known to be affected by PD [38, 39, 41]. In previous studies [63, 65], it was found that the prefrontal cortex and precentral gyrus were impaired in patients with PD when conducting motor tasks and that these areas are related to motor function. Because GB 34 was reported to be involved in motor function treatment [47], the treatment effect in the prefrontal cortex and precentral gyrus that was found in patients with PD in our study is in line with previous findings in these motor task studies [63, 65]. The prefrontal cortex is considered to induce actions in accordance with internal goals [66] and has been shown to be involved in composing and conducting voluntary behavior [67], whereas the precentral gyrus is associated with planning and execution of movement [68–70].The volume activated by ACUP in the precentral gyrus and prefrontal cortex was bigger than that by SHAM (Figure 2). This is thought that even if the precentral gyrus and prefrontal cortex were activated also by SHAM, ACUP acted on the brain stronger than SHAM. Moreover, the volume of common areas which were activated by ACUP on both patients with PD and healthy participants was significantly larger than that by SHAM (Figure 3). This result suggests that the level of ACUP activation in the brains of PD patients is similar to that of healthy participants compared against SHAM.

The limitation of this study is that we should have observed motor improvement instead of just referring to existing literature. Moreover, the patients’ mean disease duration of our participants was 2.67 years, which is a relatively benign state of PD, so the results were slightly differing from previous research and might only apply to relatively patients with early PD.

## Conclusions

Therefore, the neuroimaging results of our study suggest that in future acupuncture research; the prefrontal cortex as well as the precentral gyrus should be treated for symptoms of Parkinson’s disease and that GB 34 seems to be a suitable acupoint. Moreover, acupuncture evoked different brain activations in patients with Parkinson’s disease than in healthy participants in our study, stressing the importance of conducting acupuncture studies on both healthy participants as well as patients within the same study, in order to detect acupuncture efficacy.

## Electronic supplementary material

Additional file 1: Table S1: Demographic characteristics among patients with PD and healthy participants. (DOC 34 KB)

Additional file 2: Figure S1: Acupuncture and sham stimulations during fMRI scanning were performed at GB 34 on the right leg according to the WHO Standard Acupuncture Point Locations. (TIF 1 MB)

Additional file 3: Figure S2: The fMRI scanning paradigm. The scanning experiment started with the SHAM condition with a duration of 5 minutes. Then, structural images were acquired, lasting more than 15 minutes. After structural imaging, the ACUP condition with a duration of 5 minutes followed. (TIF 2 MB)

Additional file 4: Table S2: Neural responses among healthy participants and patients with PD during acupuncture stimulations (one-sample *t* test; with corrected cluster level *P* < 0.05). (DOC 144 KB)

Additional file 5: Figure S3: Comparison of brain activation between patients with PD and healthy participants during acupuncture stimulation. “Healthy participants > Patients with PD” indicates more activated brain activation of healthy participants compared with patients with PD. “Healthy participants < Patients with PD” indicates more activated brain activation of patients with PD compared with healthy participants (two-sample t-test; with corrected cluster level P < 0.05). The bar is the t value. (TIF 486 KB)
