# Supplementary material for: Acupuncture on GB34 activates the precentral gyrus and prefrontal cortex in Parkinson’s disease
Source: BMC Complement Altern Med. 2014 Sep 15;14:336. doi: 10.1186/1472-6882-14-336 (PMC4175221; doi:10.1186/1472-6882-14-336)
Supplement: Supplementary file 1 — Additional file 1: Table S1: Demographic characteristics among patients with PD and healthy participants. (DOC 34 KB) [file 12906_2013_1910_MOESM1_ESM.doc]

**Additional file 1: Table S1** Demographic characteristics among patients with PD and healthy participants.

|  | HP^a^ (n = 12) | PD^b^ (n = 12) |
| --- | --- | --- |
| Sex (male:female) | 6:6 | 6:6 |
| Age (years) | 55.9 ± 9.8 | 53.5 ± 10.9 |
| Disease duration (years) | - | 2.67 ± 2.3 |
| Medication duration (years) | - | 2.67 ± 2.3 |
| Hoen and Yahr stage | - | 1.5 ± 0.6 |
| UPDRS motor score^c^ | - | 7.8 ± 3.9 |
| K-MMSE^d^ | - | 27.8 ± 0.4 |
| BDI II^e^ | - | 15.36 ± 7.9 |
| EHI (right:left) ^f^ | 12:0 | 12:0 |

^a^ HP = healthy participants; ^b^ patients with PD; ^c^ UPDRS = Unified Parkinson’s Disease Rating Scale; ^d^ K-MMSE = Korean Mini-Mental State Examination; ^e^ BDI II = Beck Depression Inventory II; ^f^ EHI = Edinburgh Handedness Inventory.
