# Supplementary figures and images for: Acupuncture on GB34 activates the precentral gyrus and prefrontal cortex in Parkinson’s disease
Source: BMC Complement Altern Med. 2014 Sep 15;14:336. doi: 10.1186/1472-6882-14-336 (PMC4175221; doi:10.1186/1472-6882-14-336)

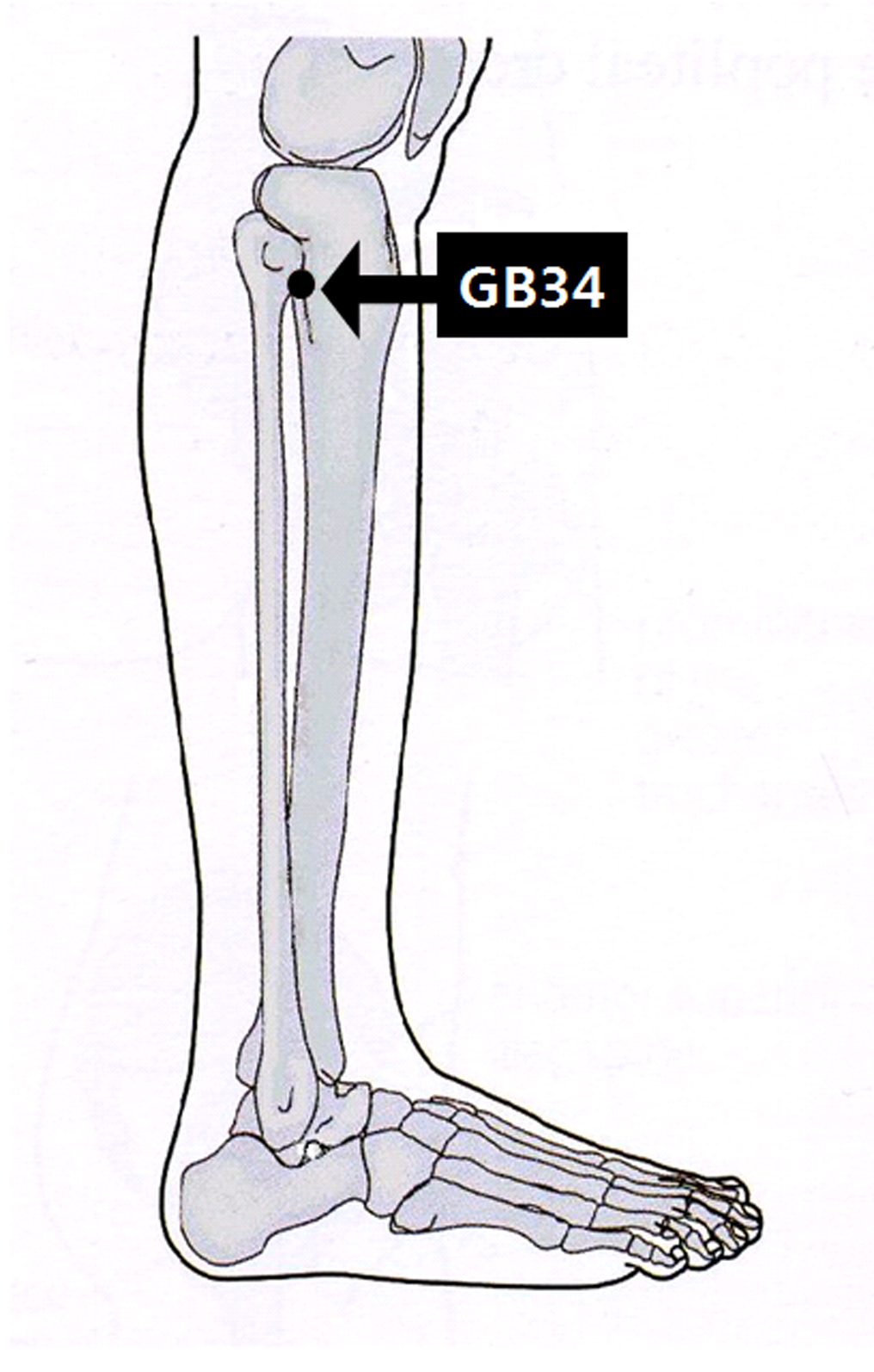

Supplement: Supplementary file 2 — Additional file 2: Figure S1: Acupuncture and sham stimulations during fMRI scanning were performed at GB 34 on the right leg according to the WHO Standard Acupuncture Point Locations. (TIF 1 MB) [file 12906_2013_1910_MOESM2_ESM.tif]

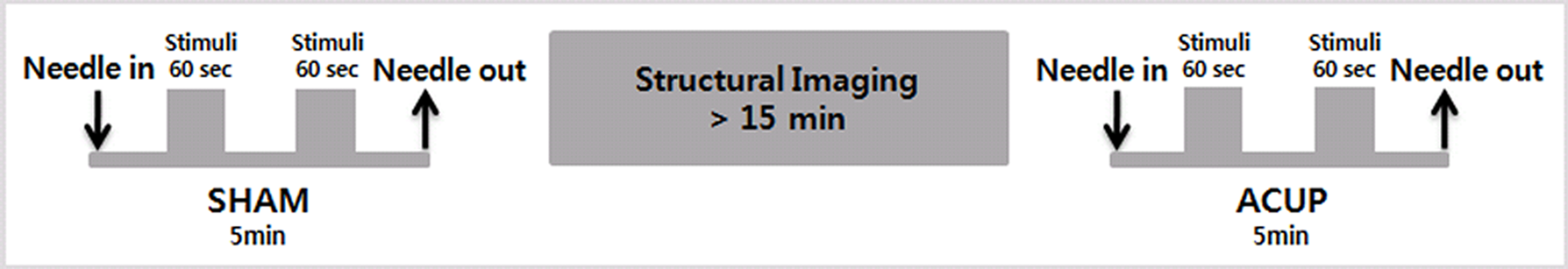

Supplement: Supplementary file 3 — Additional file 3: Figure S2: The fMRI scanning paradigm. The scanning experiment started with the SHAM condition with a duration of 5 minutes. Then, structural images were acquired, lasting more than 15 minutes. After structural imaging, the ACUP condition with a duration of 5 minutes followed. (TIF 2 MB) [file 12906_2013_1910_MOESM3_ESM.tif]

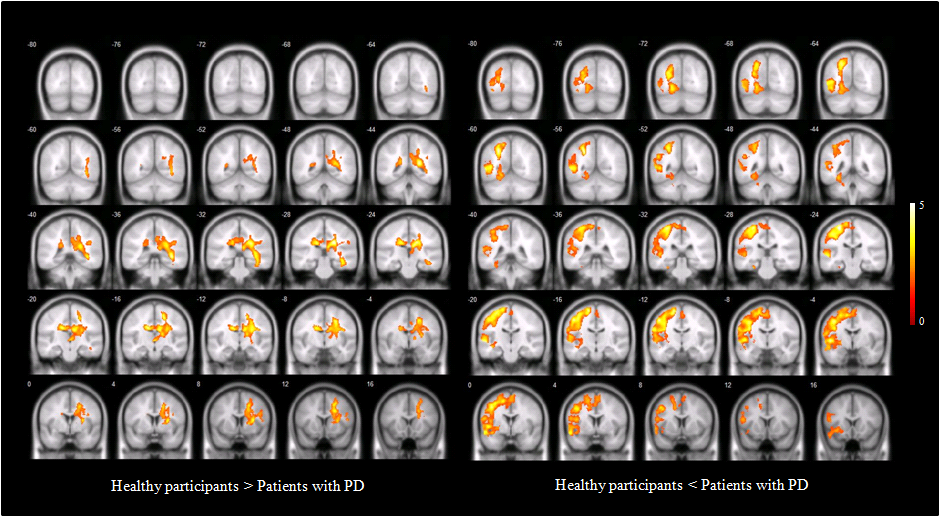

Supplement: Supplementary file 5 — Additional file 5: Figure S3: Comparison of brain activation between patients with PD and healthy participants during acupuncture stimulation. “Healthy participants > Patients with PD” indicates more activated brain activation of healthy participants compared with patients with PD. “Healthy participants < Patients with PD” indicates more activated brain activation of patients with PD compared with healthy participants (two-sample t-test; with corrected cluster level P < 0.05). The bar is the t value. (TIF 486 KB) [file 12906_2013_1910_MOESM5_ESM.tif]
