# Supplementary material for: Acupuncture on GB34 activates the precentral gyrus and prefrontal cortex in Parkinson’s disease
Source: BMC Complement Altern Med. 2014 Sep 15;14:336. doi: 10.1186/1472-6882-14-336 (PMC4175221; doi:10.1186/1472-6882-14-336)
Supplement: Supplementary file 4 — Additional file 4: Table S2: Neural responses among healthy participants and patients with PD during acupuncture stimulations (one-sample t test; with corrected cluster level P < 0.05). (DOC 144 KB) [file 12906_2013_1910_MOESM4_ESM.doc]

**Additional file 4: Table S2** Neural responses among healthy participants and patients with PD during acupuncture stimulations (one-sample *t* test; with corrected cluster level *P* < 0.05).

|  | | |  | **Healthy participants** | | | | |  |  | | |  |  | **Patients with PD** | | | |  |  | | |  |
| --- | --- | --- | --- | --- | --- | --- | --- | --- | --- | --- | --- | --- | --- | --- | --- | --- | --- | --- | --- | --- | --- | --- | --- |
| Cerebral area | | |  | Coordinates anatomical location | | | | Statistical values |  | Coordinates anatomical location | | | Statistical values |  | Coordinates anatomical location | | | Statistical values |  | Coordinates anatomical location | | | Statistical values |
| Brain region | L/R | Brodmann area |  | *x* | *y* | | *z* | *t* value |  | *x* | *y* | *z* | *t* value |  | *x* | *y* | *z* | *t* value |  | *x* | *y* | *z* | *t* value |
|  |  |  |  | ***Sham*** | | | |  |  | ***Acupuncture*** | | |  |  | ***Sham*** | | |  |  | ***Acupuncture*** | | |  |
| *Frontal lobe* |  |  |  |  | |  |  |  |  |  |  |  |  |  |  |  |  |  |  |  |  |  |  |
| Superior frontal gyrus | Right | 6 |  | - | | - | - | - |  | - | - | - | - |  | - | - | - | - |  | 19 | -4 | 67 | 4.15 |
| Inferior frontal gyrus | Right | 44/45 |  | 49 | | 5 | 15 | 5.95 |  | - | - | - | - |  | 49 | 2 | 20 | 5.48 |  | 51 | 20 | 9 | 4.52 |
| Precentral gyrus | Right | 6/9/44 |  | 43 | | 3 | 11 | 6.11 |  | - | - | - | - |  | 51 | 1 | 14 | 5 |  | 38 | 14 | 35 | 3.87 |
| Paracentral lobule | Right | 5 |  | - | | - | - | - |  | - | - | - | - |  | 4 | -37 | 57 | 3.59 |  | - | - | - | - |
| Superior frontal gyrus | Left | 6 |  | - | | - | - | - |  | - | - | - | - |  | -13 | -6 | 67 | 5.51 |  | - | - | - | - |
| Medial frontal gyrus | Left | 6 |  | - | | - | - | - |  | - | - | - | - |  | - | - | - | - |  | -11 | -13 | 59 | 3.65 |
| Middle frontal gyrus | Left | 6/46 |  | - | | - | - | - |  | - | - | - | - |  | -47 | 39 | 14 | 3.9 |  | -35 | -6 | 48 | 3.58 |
| Inferior frontal gyrus | Left | 44/46 |  | - | | - | - | - |  | - | - | - | - |  | -47 | 35 | 12 | 3.87 |  | -51 | 4 | 20 | 4.14 |
| Precentral gyrus | Left | 4/6/44 |  | - | | - | - | - |  | - | - | - | - |  | -53 | 5 | 9 | 6.39 |  | -17 | -20 | 58 | 4.13 |
|  |  |  |  |  | |  |  |  |  |  |  |  |  |  |  |  |  |  |  |  |  |  |  |
| *Parietal lobe* |  |  |  |  | |  |  |  |  |  |  |  |  |  |  |  |  |  |  |  |  |  |  |
| Inferior Parietal lobule | Right | 40 |  | - | | - | - | - |  | - | - | - | - |  | 50 | -31 | 29 | 4.11 |  | - | - | - | - |
| Superior parietal lobule | Left | 7 |  | - | | - | - | - |  | - | - | - | - |  | -17 | -49 | 61 | 7.34 |  | - | - | - | - |
| Supramarginal gyrus | Left | 40 |  | - | | - | - | - |  | - | - | - | - |  | - | - | - | - |  | -55 | -36 | 32 | 5.5 |
| Inferior Parietal lobule | Left | 40 |  | - | | - | - | - |  | - | - | - | - |  | -50 | -26 | 28 | 4.19 |  | -57 | -39 | 26 | 4.9 |
| Postcentral gyrus | Left | 3/5/43 |  | - | | - | - | - |  | - | - | - | - |  | -20 | -38 | 63 | 5 |  | -57 | -12 | 17 | 4.76 |
| Precentral gyrus | Left | 4 |  | - | | - | - | - |  | - | - | - | - |  | - | - | - | - |  | -30 | -29 | 57 | 4.73 |
| Precuneus | Left | 7 |  | - | | - | - | - |  | - | - | - | - |  | -15 | -49 | 43 | 3.89 |  | - | - | - | - |
|  |  |  |  |  | |  |  |  |  |  |  |  |  |  |  |  |  |  |  |  |  |  |  |
| *Temporal lobe* |  |  |  |  | |  |  |  |  |  |  |  |  |  |  |  |  |  |  |  |  |  |  |
| Superior temporal gyrus | Right | 22 |  | - | | - | - | - |  | - | - | - | - |  | 53 | 5 | 6 | 4.29 |  | 49 | 2 | 3 | 5.33 |
| Superior temporal gyrus | Left | 22/42 |  | - | | - | - | - |  | - | - | - | - |  | -61 | -31 | 17 | 6.57 |  | -55 | 6 | 4 | 4.61 |
| Middle temporal gyrus | Left | 21/37 |  | - | | - | - | - |  | - | - | - | - |  | -49 | 58 | 3 | 5.52 |  | - | - | - | - |
| Inferior temporal gyrus | Left | 37 |  | - | | - | - | - |  | - | - | - | - |  | -46 | -52 | 2 | 5.11 |  | - | - | - | - |
| Angular gyrus | Left | 39 |  | - | | - | - | - |  | - | - | - | - |  | - | - | - | - |  | -28 | -59 | 36 | 4.6 |
| Transverse temporal gyrus | Left | 4 |  | - | | - | - | - |  | - | - | - | - |  | -51 | -25 | 14 | 5.56 |  | - | - | - | - |
|  |  |  |  |  | |  |  |  |  |  |  |  |  |  |  |  |  |  |  |  |  |  |  |
| *Sub-lobar* |  |  |  |  | |  |  |  |  |  |  |  |  |  |  |  |  |  |  |  |  |  |  |
| Putamen | Right |  |  | 17 | | -3 | 13 | 7.16 |  | 19 | 5 | 9 | 7.75 |  | 30 | -15 | 2 | 3.56 |  | 25 | 4 | 3 | 3.58 |
| Thalamus | Right |  |  | 12 | | -8 | 9 | 5.95 |  | 14 | 16 | 16 | 6.52 |  | - | - | - | - |  | - | - | - | - |
| Caudate body | Right |  |  | - | | - | - | - |  | 15 | 0 | 21 | 4.69 |  | - | - | - | - |  | - | - | - | - |
| Insula | Right | 13 |  | 41 | | 1 | 14- | 7.85 |  | 29 | 23 | 14 | 4.58 |  | 52 | -23 | 25 | 5.75 |  | 45 | 11 | 3 | 4.33 |
| Lateral globus pallidus | Right |  |  | - | | - | - | - |  | 25 | -9 | 0 | 4.5 |  | - | - | - | - |  | - | - | - | - |
| Claustrum | Right |  |  | 30 | | 2 | 18 | 4.7 |  | - | - | - | - |  | 35 | -1 | -2 | 5.92 |  | - | - | - | - |
| Putamen | Left |  |  | - | | - | - | - |  | - | - | - | - |  | - | - | - | - |  | -23 | -6 | 7 | 3.57 |
| Thalamus | Left |  |  | - | | - | - | - |  | -1 | -21 | 6 | 4.55 |  | - | - | - | - |  | - | - | - | - |
| Insula | Left | 13 |  | - | | - | - | - |  | - | - | - | - |  | -53 | -39 | 21 | 7.85 |  | - | - | - | - |
| Claustrum | Left |  |  | - | | - | - | - |  | - | - | - | - |  | -36 | -6 | -4 | 4.29 |  | -38 | -19 | -7 | 3.51 |
|  |  |  |  |  | |  |  |  |  |  |  |  |  |  |  |  |  |  |  |  |  |  |  |
| *Limbic lobe* |  |  |  |  | |  |  |  |  |  |  |  |  |  |  |  |  |  |  |  |  |  |  |
| Posterior cingulate | Right | 23 |  | - | | - | - | - |  | 6 | -26 | 22 | 4.7 |  | - | - | - | - |  | - | - | - | - |
| Cingulate gyrus | Right | 24 |  | - | | - | - | - |  | 19 | 6 | 42 | 4.6 |  | - | - | - | - |  | - | - | - | - |
|  |  |  |  |  | |  |  |  |  |  |  |  |  |  |  |  |  |  |  |  |  |  |  |
| *Occipital lobe* |  |  |  |  | |  |  |  |  |  |  |  |  |  |  |  |  |  |  |  |  |  |  |
| Middle occipital gyrus | Left | 37 |  | - | | - | - | - |  | - | - | - | - |  | -47 | -62 | -10 | 5.16 |  | - | - | - | - |
|  |  |  |  |  | |  |  |  |  |  |  |  |  |  |  |  |  |  |  |  |  |  |  |
| *Cerebellum* | Right |  |  | - | | - | - | - |  | 44 | -64 | 39 | 6.17 |  | - | - | - | - |  | - | - | - | - |
| Cluster size |  |  |  | 507 | |  |  |  |  | 12914 |  |  |  |  | 7361  3489  4023 |  |  |  |  | 8304  2407 |  |  |  |

The table describes the location of the peak voxel and the corresponding brain regions and Brodmann areas comprised by the cluster. Results are reported if cluster level corrected *P* < 0.05 and t value >3.5. The voxel size is 2.4 x 2.4 x 4.5 mm.
